# Potential therapeutic effects of endothelial cells trans-differentiated from Wharton’s Jelly-derived mesenchymal stem cells on altered vascular functions in aged diabetic rat model

**DOI:** 10.1186/s13098-020-00546-y

**Published:** 2020-05-11

**Authors:** Shaimaa M. Motawea, Rasha I. Noreldin, Yahya M. Naguib

**Affiliations:** 1 Clinical Physiology Department, Faculty of Medicine, Menoufia University, Menoufia, Egypt; 2 Clinical Pathology Department, Faculty of Medicine, Menoufia University, Menoufia, Egypt; 3 Physiology Department, College of Medicine and Medical Sciences, Arabian Gulf University, Manama, Bahrain

**Keywords:** Endothelial cells, Mesenchymal stem cells, Aging, Diabetes mellitus, Hypertension

## Abstract

**Background:**

Diabetes mellitus in elderly represents an exceptional subset in the population vulnerable to cardiovascular events. As aging, diabetes mellitus and hypertension share common pathways, an ideal treatment should possess the ability to counter more than one of, if not all, the underlying mechanisms. Stem cells emerged as a potential approach for complicated medical problems. We tested here the possible role of trans-differentiated endothelial cells (ECs) in the treatment of diabetes mellitus in old rats.

**Methods:**

Mesenchymal stem cells where isolated from umbilical cord Wharton’s Jelly and induced to differentiate into endothelial like-cells using vascular endothelial growth factor-enriched media. Thirty aged male Wistar albino rats were used in the present study. Rats were divided (10/group) into: control group (18–20 months old, weighing 350–400 g, received single intraperitoneal injection as well as single intravenous injection via tail vein of the vehicles), aged diabetic group (18–20 months old, weighing 350–400 g, received single intraperitoneal injection of 50 mg/kg streptozotocin, and also received single intravenous injection of saline via tail vein), and aged diabetic + ECs group (18–20 months old, weighing 350–400 g, received single intraperitoneal injection of 50 mg/kg streptozotocin, and also received single intravenous injection of 2*10^6^ MSC-derived ECs in 0.5 ml saline via tail vein) groups. Assessment of SBP, aortic PWV, and renal artery resistance was performed. Serum levels of ET1, ANG II, IL-6, TNF-α, MDA, ROS, and VEGF were evaluated, as well as the aortic NO tissue level and eNOS gene expression. Histopathological and immunostaining assessments of small and large vessels were also performed.

**Results:**

Induction of diabetes in old rats resulted in significant increase in SBP, aortic PWV, renal artery resistance, and serum levels of ET1, ANG II, IL-6, TNF-α, MDA, ROS, and VEGF. While there was significant decrease in aortic NO tissue level and eNOS gene expression in the aged diabetic group when compared to aged control group. ECs treatment resulted in significant improvement of endothelial dysfunction, inflammation and oxidative stress.

**Conclusion:**

We report here the potential therapeutic role of trans-differentiated ECs in aged diabetics. ECs demonstrated anti-inflammatory, antioxidant, gene modifying properties, significantly countered endothelial dysfunction, and improved vascular insult.

## Background

Aging is the main risk factor for atherosclerosis and, therefore, for ischemic heart disease. Apart from age-associated changes of the vascular wall, which includes luminal enlargement, increased intimal and medial thickness, and vascular stiffness, endothelial function declines with age [1]. This is most obvious from the attenuation of endothelium-dependent dilator responses, which is a consequence of the alteration in the expression and/or activity of the endothelial NO synthase (eNOS), upregulation of the inducible NO synthase (iNOS), and increased oxidative stress. Aging is also associated with a reduction in the regenerative capacity of the endothelium and endothelial senescence, which is characterized by an increased rate of endothelial cell apoptosis [2]. Diabetes mellitus is associated with an increased risk of cardiovascular diseases (CVD), even in the presence of strict glycemic control. Many studies suggest that both diabetes and impaired glucose tolerance cause a combination of endothelial dysfunctions, which may affect the anti-atherogenic role of the vascular endothelium [3]. The majority of deaths that occur in diabetic patients are due to vascular dysfunction. Studies have shown that endothelial dysfunction, as represented by impaired endothelium-dependent nitric oxide (NO)-mediated relaxation, occurs in diabetes. Mechanisms underlying this endothelial dysfunction could include decreased activity and/or expression of eNOS or increased degradation of NO secondary to enhanced superoxide production [4].

The inside of the blood vessels is lined with the vascular endothelium; a thin layer that helps in the regulation of blood coagulation, immune responses and vascular tone [5]. Endothelial dysfunction means that the capacity of the endothelium to perform these functions is reduced, rendering the blood vessels functioning improperly [6]. In endothelial dysfunction, there is impairment of the endothelial-dependent dilatation (EDD), adherence of leucocytes, activation of platelets, mitogenesis, increased oxidative stress, vascular inflammation and thrombosis [7]. Endothelial dysfunction can be a significant predictor of coronary artery disease and atherosclerosis; chronic disorders which can cause stroke or heart attack [8].

Cell therapy is the use of living cells to maintain, restore or enhance the functions of organs and tissues [9]. Stem cells are characterized by their capacity for cell renewal, which leads to their differentiation and formation of one or more mature tissues [10]. Mesenchymal stem cells (MSCs) are defined as adherent cells which possess a proliferative potential and an ability to differentiate in vitro into osteogenic, chondrogenic, myogenic and adipogenic lineages [11]. MSCs have been also demonstrated to differentiate into endothelial cells [12]. MSCs can be isolated from adipose tissue, umbilical cord blood, and various foetal tissues such as the placenta, amniotic fluid and amniotic membrane [13]. Many studies have shown that Wharton’s Jelly in the human umbilical cord is also a rich source of primitive MSCs [14]. Therefore, we hypothesised that transplanted ECs could counteract the altered vascular functions in aged diabetic rats.

## Materials and methods

### Ethics statement

This study conforms to the principles outlined in the Declaration of Helsinki. Ethical approval was received from the Ethics Committee of the Faculty of Medicine of Menoufia University and Menoufia University Teaching Hospital (Egypt). Informed written consent was obtained from all patients.

### Isolation and culture of human mesenchymal stem cells isolated from Wharton’s Jelly (hWMSCs)

Umbilical cords were aseptically collected from 10 healthy full-term pregnancies, at Menoufia University Teaching Hospital (Egypt). hWMSCs were isolated as described previously with trivial modifications. Umbilical cords were cut into 2 to 3 mm pieces and vessels were stripped manually from those cord segments. Wharton’s Jelly was digested with collagenase 10 mg/mL (37 ℃ for 4 h). Mesenchymal cells were recovered, centrifuged (1000 g for 30 min) and suspended in fresh Dulbecco’s Modified Eagle’s (DMEM) medium including cord blood serum (100 µl/ml), l-glutamine (100 μg/mL), penicillin (100 U/ml), streptomycin (100 μg/ml streptomycin) and fungizone (0.25 μg/mL). Cultures were maintained in a humidified incubator with 5% CO_2_ at 37 ℃ for 10 days, until hWMSCs colonies were observed. Adherent cells were detached using a trypsin–EDTA solution [15].

### Flow cytometry

Cell characterization by flow cytometry was carried out as previously described [16]. Briefly, hWMSCs in culture were trypsinized (0.25% trypsin/1 mM EDTA) and then incubated in the dark for 30 min with CD44 and CD34 monoclonal antibodies (R&D System, USA) conjugated with phycoerythrin (PE). 100 μl of cell suspension was added to 10 μl of CD44-PE and CD34-PE. After incubation cells were gated out on CD44 and CD34 expression an argon laser FACS Calibur (Becton–Dickinson, USA), at the Laboratory of Clinical Pathology Department, Menoufia University Teaching hospitals.

### Endothelial differentiation

Endothelial differentiation was performed as described previously with some modifications [17]. hWMSCs were incubated in 35 mm plates in primary culture medium supplemented with 50 ng/ml vascular endothelial growth factor (VEGF, R&D System, USA) for 8 consecutive days. Endothelial phenotype was confirmed through flow-cytometric analysis of the surface markers CD34-PE as described in the previous paragraph. On the 8th day of differentiation, medium was aseptically removed using a Pasteur pipette. 1 ml of trypsin- EDTA solution was added to the 35 mm dish. The dish was gently shaken and taped on the sides to ensure complete detachment of the cells. After complete detachment, 1 ml DMEM containing 1% CBS was added and the medium was pipetted several time to break up any cell clumps. The cell suspension was then transferred into a 5 ml Eppendorf conical tube and centrifuged at 1800 rpm for 10 min. The supernatant was removed and cells were suspended in isotonic saline at a density of 4 × 10^6^ cells/ml.

### Animals and experimental design

All experimental procedures were conducted in adherence to the Guiding Principles in the Use and Care of Animals published by the National Institutes of Health (NIH Publication No 85–23, Revised 1996). Animal care and use was approved by Ethics Committee, Menoufia Faculty of Medicine, Egypt. 30 aged male Wistar albino rats were purchased from a local experimental animals providing facility and recruited for the present study. Animals were kept for 10 days prior to the start of the study to allow proper acclimatization. The animals were fed standard laboratory chow and allowed free access to water in an air-conditioned room with a 12 h light–dark cycle. Rats were then divided randomly into the following groups (10 rats per group):

1. Control group: 18–20 months old, weighing 350–400 g received a single intraperitoneal injection of isotonic saline. Six weeks later, they received a single intravenous injection of isotonic saline via tail vein
2. Aged group: 18–20 months old, weighing 350–400 g, received a single intraperitoneal injection of 50 mg/kg streptozotocin (Sigma-Aldrich, St Louis, MO, USA). Diabetes mellitus was confirmed forty-eight hours after streptozotocin injection by measuring fasting blood glucose level. Rats with fasting blood glucose levels ≥ 200 mg/dl were considered diabetic. 6 weeks later rats received a single intravenous injection of isotonic saline via tail vein.
3. Aged diabetic + mesenchymal cell-derived endothelial cells group: (18–20 months old, weighing 350–400 g, received single intraperitoneal injection of 50 mg/kg streptozotocin. Rats with fasting blood glucose levels ≥ 200 mg/dl measured forty-eight hours following streptozotocin injection were considered diabetic. 6 weeks later rats received a single intravenous injection of 2*10^6^ ECs in 0.5 ml isotonic saline via tail vein.

Four weeks following the tail vein injection of isotonic saline or ECs, all rats were subjected to:

### Measurement of systolic blood pressure

Systolic blood pressure was determined in the rats by means of a rat-tail pressure detecting equipment (Harvard apparatus Ltd, Aden Berge, England) connected to a pneumatic transducer (Harvard U.K.). Changes in pressure were recorded via a physiograph (MK III-S, Narco BioSystem, USA).

### Doppler studies

Evaluation of aortic pulse wave velocity (PWV) and renal artery resistance (RAR) was done using pulsed doppler flowmeter (Hadeco, Hayashi Denki Co. Ltd., Japan). Briefly, rats were anesthetized via intraperitoneal injection of sodium thiopental (STP, 60 mg/kg), and a midline abdominal incision was made exposing the aorta and the left renal arteries. The tip of the probe was filled with coupling gel, and then the probe was placed over the blood vessel until stable signals could be recorded. Aortic PWV was assessed using doppler ultrasound and the equation method as described previously with some modifications [18].

### Blood sample collection

At the end of the experiments, all rats were fasted overnight. Rats were then anaesthetised using STP (as mentioned vid supra). The chest wall was opened by extending the midline incision previously performed for the doppler study to facilitate blood sample collection and excision of the aorta for the tissue homogenate preparation and PCR experiments. Blood samples were collected via cardiac puncture, left to clot for 10–15 min and then centrifuged at 3000 rpm for another 15 min. Serum samples were stored at − 20 °C for subsequent analysis of different biomarkers. All rats were then scarified by cervical dislocation.

### Preparation of aortic tissue homogenate

A midline incision was made to open the chest and abdominal walls and expose the aorta. The aorta was excised and cross-chopped into fine slices using a surgical scalpel. Rat aorta was homogenized in ice cold phosphate buffer (pH 7.4), suspended in chilled 0.25 M sucrose solution, and rapidly blotted on a filter paper. Mincing and homogenization of tissue were performed to release soluble proteins in ice-cold Tris hydrochloride buffer (10 mM, pH 7.4). Tissue homogenate was centrifuged at 7000 rpm for 20 min and the supernatant was collected and stored at − 20 °C for subsequent estimation of NO.

### Biochemical analysis

Serum levels of interleukin 6 (IL-6), tumour necrosis factor alpha (TNF-α) (Quantikine^®^ ELISA, R&D Systems Inc., MN, USA), endothelin-1 (ET-1) (Immuno-Biological Laboratories Co. Ltd., Takasaki-Shi, Gunma, Japan), angiotensin II (AGN-II) (Sigma-Aldrich, St Louis, MO, USA), and advanced glycation end products (AGEs) (MyBioSource, USA) were determined by quantitative sandwich enzyme immunoassay technique using an automatic optical reader (SUNRISE Touchscreen, TECHAN, Salzburg, Austria). Serum levels of malondialdehyde (MDA) (QuantiChrom™, BioAssay Systems, USA), and reactive oxygen species (ROS) (Elabscience^®^, USA) were determined by routine kinetic and fixed rate colorimetric methods on a Jenway Genova autoanalyser (UK). Tissue level of nitric oxide (NO) (QuantiChrom™, BioAssay Systems, USA) was determined by quantitative sandwich enzyme immunoassay technique.

### Quantification of eNOS mRNA

The thoracic aorta was isolated and cut into 12 mm segments. Total RNA was extracted from rat aorta using TRI reagent (Sigma-Aldrich, UK). Extracted RNAs were reverse transcribed using the high capacity RNA-to-cDNA kit (Applied Biosystems, Foster City, CA, USA) according to the manufacturer’s instructions. Real-time RT-PCR was performed using a Biosystem 7300 (Applied Biosystems, CA, USA). To quantify changes in gene expression, the comparative Ct method was used to calculate the relative-fold changes normalized relative to the housekeeping gene GAPDH. The gene specific primers for eNOS were: 5′-ACT GCG TCG CTT CAT TAG GT-3′ (forward) and 5′-TAG GCA AGC GCT TTA CCA CT-3′ (reverse), while primers for GAPDH were: 5′-TGG GTG TGA ACC ACA AGA AA-3′ (forward) and 5′-GTG GCA GTG ATG ACA TGG AC-3′ (reverse) for rat GAPDH. Results are shown as the mean of three samples, with each sample assayed in duplicate.

### Haematoxylin and Eosin (H&E) stain

Specimen from both thoracic aortas and cardiac muscles of the aged diabetic ECs treated and non-treated rats were fixed in 10% formol saline for 4–6 days. The specimens were thoroughly washed in tap water and then dehydrated in graded ethanol solutions. After that, the specimens were cleared in xylene depending on the size of the specimen (guided by inspection at 5 min intervals). The specimens were soaked in soft paraffin wax at 60 °C for 2 h, and then in hard paraffin wax at room temperature. Next, tissue blocks were cut into 5 micron-thick sections using rotator microtome. Tissue sections were dipped in a warm water-bath, picked up on clean slides, and placed on hot plate for 2 min. Finally, tissue sections were stained with haematoxylin and eosin.

### CD31 immunostaining

Antihuman primary antibody to CD31 (Human CD31/PECAM-1 Antibody, R&D Systems, USA) was used for the immunostaining experiments. The tissue sections were incubated with the anti-CD31 antibody overnight at 4 °C. The binding of the primary antibody was observed using a commercial avidinbiotin-peroxidase detection system recommended by the manufacturer (DAKO, Carpenteria, USA). Finally, the slides were stained with diaminobenzene (DAB).

### Statistical analysis

Results are expressed as mean ± standard deviation (SD). Kolmogorov–Smirnov test was performed on all data sets to ensure normal distribution (p > 0.5). Analyses of Variances (ANOVA) with Tukey’s honesty significant difference (HSD) tests were used for statistical analysis using Origin^®^ software. The probability of chance (p values) values < 0.05 were considered significant.

## Results

**Fig. 1:**
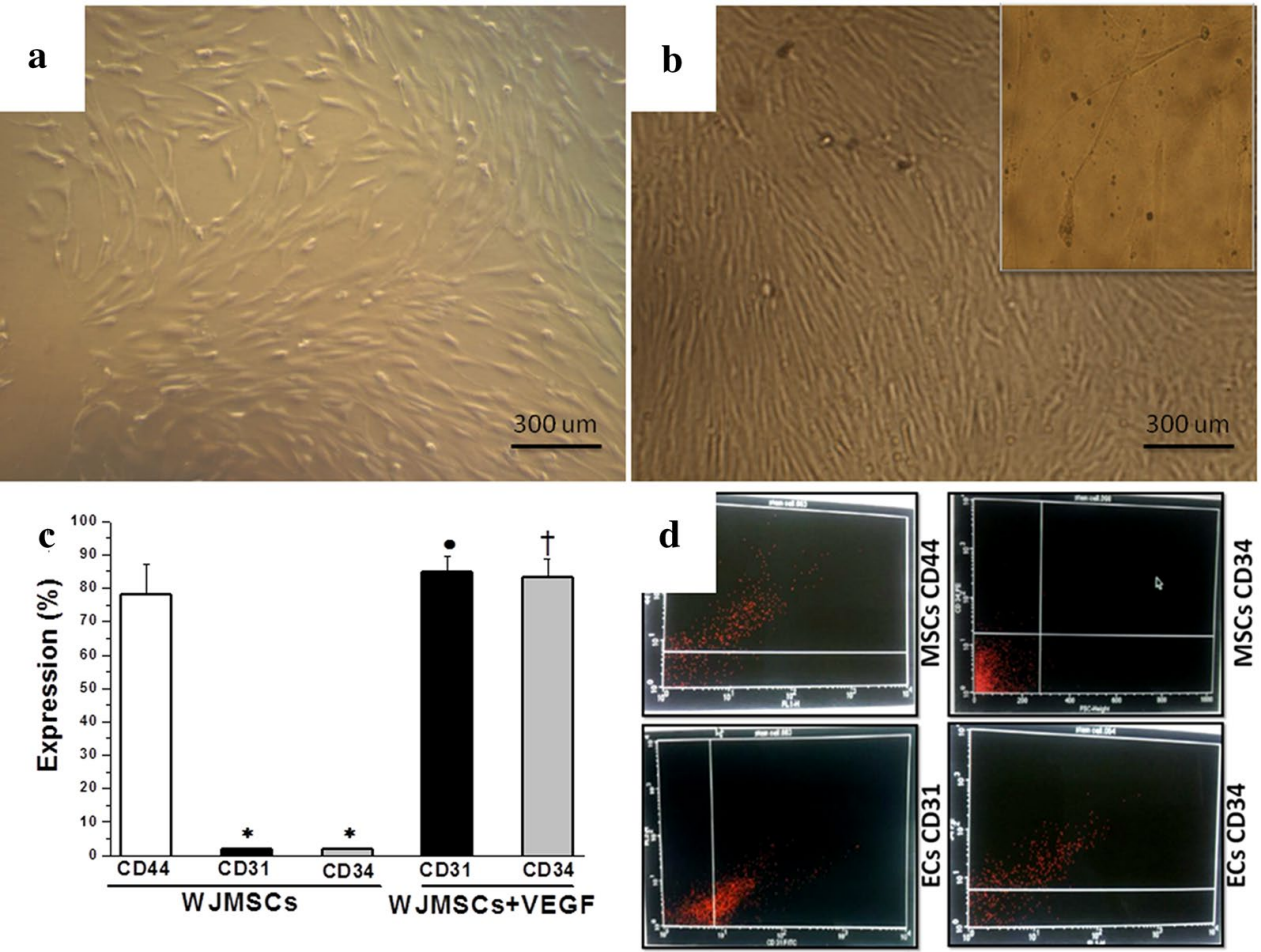
Separation and differentiation of WJ-MSCs into ECs. **a** Inverted microscope image of Wharton’s Jelly-derived MSCs showing adherent fibroblasts-like cells (X 100). **b** Inverted microscope image of ECs differentiated from MSCs following VEGF application (X 100, insert X 400). **c** Expression of CD44, CD31 and CD 34 in WJ-MSCs, and expression of CD31 and CD34 10 days after VEGF application. (*Significant (p < 0.05), N = 10). **d** Representative flow cytometry analysis of CD44, CD31 and CD 34 in WJ-MSCs before and after VEGF application. (Significant = p < 0.05. *Significant when compared to CD44 expression. ^•^Significant when compared to CD31 expression in WJ-MSCs. ^†^Significant when compared to CD34 expression in WJ-MSCs. N = 10)

The onset of fibroblast like cells formation could be observed approximately 5 to 7 days following the first seeding of Wharton’s Jelly derived mesenchymal stem cells (WJ-MSCs). It showed adherent mesenchymal-like cells that grew as spindle-shaped cells. Eventually, multi-polar fibroblastoid cells developed. These cells gradually reached 60–70% confluence in 14 days (Fig. 1a) Induction by VEGF resulted in gradual morphological changes ending by the formation of endothelial-like cells at day 10 (Fig. 1b). Immunophenotype analysis demonstrated that WJ-MSCs showed significant positive expression of CD44 when compared to CD34 (73.14 ± 0.1.83 vs 1.1 ± 0.13%, p < 0.001). Following induction with VEGF, the induced cells showed statistically significant increased positive expression of CD34 (77.14 ± 2.41 vs 1.1 ± 0.13%, p < 0.001), when compared to the non-induced WJ-MSCs (Fig. 1c).

**Fig. 2:**
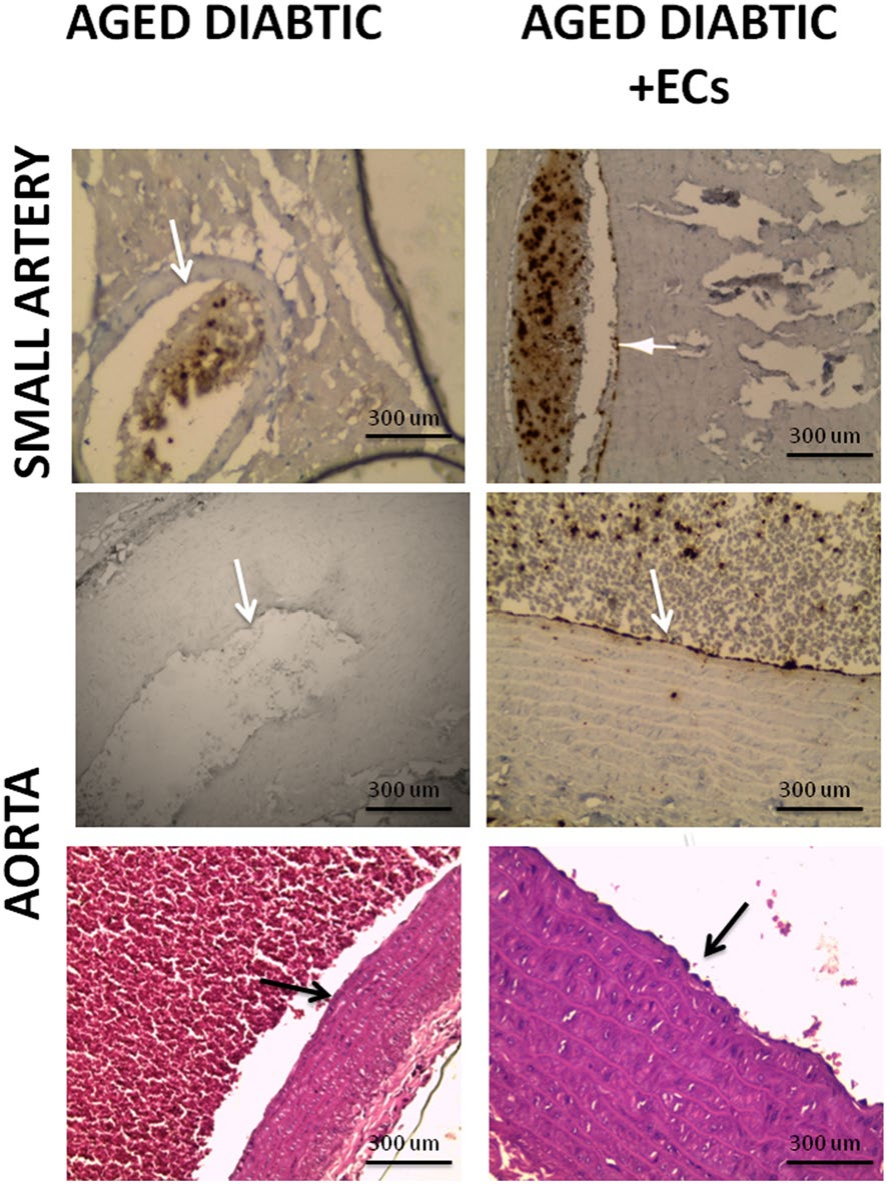
ECs homing to vascular endothelium. Left upper panel is a representative photomicrograph of a section in the cardiac muscle of aged diabetic rats showing negative CD31 immunostaining (X200). Right upper panel is a representative photomicrograph of a section in the cardiac muscle of aged diabetic + ECs treated rats showing positive CD31 immunostaining (X200). Left middle panel is a representative photomicrograph of a section in the thoracic aorta of aged diabetic rats showing negative CD31 immunostaining (X200). Right middle panel is a representative photomicrograph of a section in the thoracic aorta of aged diabetic + ECs treated rats showing positive CD31 immunostaining (X400). Left lower panel shows representative photomicrograph of H&E stained section from the thoracic aorta in the aged diabetic group (X200). Right lower panel shows representative photomicrograph of H&E stained section from the thoracic aorta in the aged diabetic + ECs group (X400)

Immunostaining using the anti-human CD31 antibody revealed successful homing of the transplanted ECs to the endothelial lining of both small vessels and the aorta (Fig. 2 right upper and middle panels) when compared to the non-treated aged diabetic rats (Fig. 2 left upper and middle panels). Histopathological evaluation of sections from the thoracic aorta of aged diabetic rats demonstrated partially denuded and ulcerated endothelium. The sub-endothelial layer showed lymphocytic infiltration and vacuolization. The media showed uneven distribution of collagen and elastic fibers, focal attenuation, and abnormal contour and fragmentation of the smooth muscle nuclei. The adventitia showed lymphocytic infiltration (Fig. 2 left lower panel). Sections from the thoracic aorta of diabetic aged rats treated with ECs demonstrated patent lumen with almost normal endothelial lining resting on sub-endothelial tissue with trivial lymphocytic infiltration. The media revealed average contour of the smooth muscle, and the collagen and elastic fibers are regularly distributed (Fig. 2 right lower panel).

**Fig. 3:**
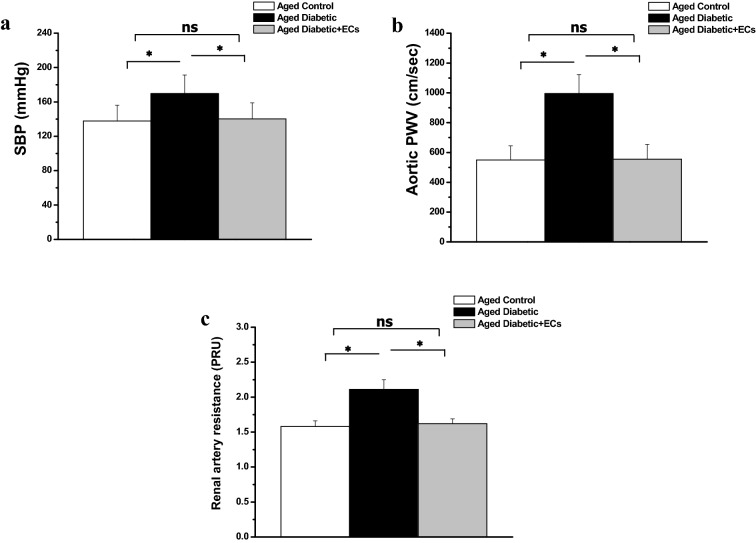
Effect of MSCs-derived ECs on vascular compliance in diabetic aged rats. **a** Systolic blood pressure (SBP) in aged control (white column), aged diabetic (black column) and aged diabetic + ECs treated (grey column) groups. **b** Aortic pulse wave velocity (PWV) in aged control (white column), aged diabetic (black column) and aged diabetic + ECs treated (grey column) groups. **c** Renal artery resistance (RAR) in aged control (white column), aged diabetic (black column) and aged diabetic + ECs treated (grey column) groups. (*Significant (p < 0.05), ns = non significant. Number of rats = 10/group)

Systolic blood pressure (SBP) increased significantly in the aged diabetic rats when compared to the aged control rats group (169.71 ± 21.53 vs 137.82 ± 18.38 mmHg, p < 0.05). SBP decreased significantly in the aged diabetic + ECs rats when compared to the aged diabetic rats group (140.18 ± 18.71 mmHg, p < 0.05). Aortic pulse wave velocity (PWV) increased significantly in the aged diabetic rats when compared to the aged control group (995.16 ± 127.31 vs 549.69 ± 94.72 cm/sec, p < 0.05). Aortic PWV decreased significantly in the aged diabetic + ECs rats when compared to the aged diabetic rats (554.87 ± 99.48 cm/sec, p < 0.05). Renal artery resistance (RAR) increased significantly in the aged diabetic rats when compared to the aged control rats (2.11 ± 0.14 vs 1.58 ± 0.08 PRU, p < 0.05). RAR decreased significantly in the aged diabetic + ECs rats when compared to the aged diabetic group (1.62 ± 0.07 PRU, p < 0.05). There was no significant difference in SBP, PWV and RAR between the diabetic + ECs and the aged control groups (p > 0.05) (Fig. 3).

**Fig. 4:**
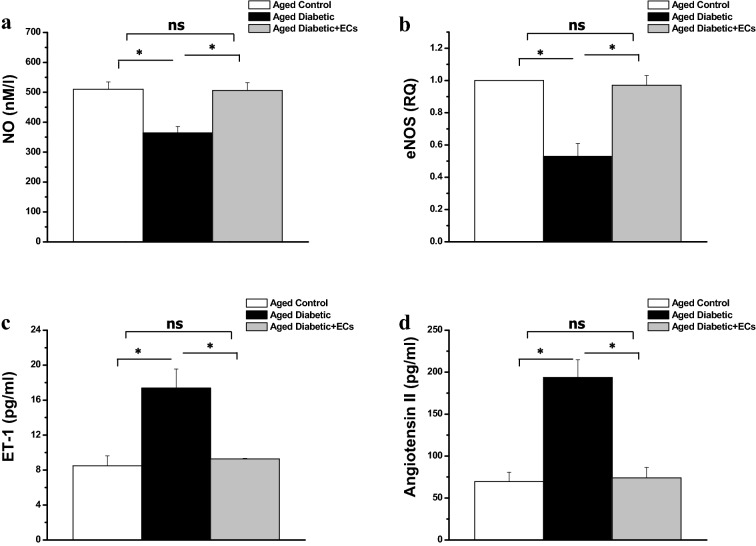
MSCs-derived ECs counter vasoconstriction/vasodilatation imbalance in diabetic aged rats. **a** Aortic tissue level of nitric oxide (NO) in aged control (white column), aged diabetic (black column) and aged diabetic + ECs treated (grey column) groups. **b** Endothelial nitric oxide synthase (eNOS) gene expression in aged control (white column), aged diabetic (black column) and aged diabetic + ECs treated (grey column) groups. **c** Serum level of endothelin 1 (ET-1) in aged control (white column), aged diabetic (black column) and aged diabetic + ECs treated (grey column) groups. **d** Serum level of angiotensin II in aged control (white column), aged diabetic (black column) and aged diabetic + ECs treated (grey column) groups. (*Significant (p < 0.05), ns = non significant. Number of rats = 10/group)

Tissue level of nitric oxide (NO) in the aorta was significantly lower in the aged diabetic when compared to the aged control rats (364 ± 21.72 vs 510 ± 24.72 nM/l, p < 0.05). Tissue NO increased significantly in the aged diabetic + ECs rats when compared to the aged diabetic group (506 ± 26.32 nM/l, p < 0.05). The aortic gene expression of the endothelial nitric oxide synthase (eNOS) was downregulated significantly in the aged diabetic when compared to the aged rats (0.53 ± 0.08 vs 1 RQ, p < 0.05). eNOS gene expression was significantly higher in the aged diabetic + ECs rats when compared to the aged diabetic rats group (0.97 ± 0.06 RQ, p < 0.05). There was no significant difference in tissue NO level or eNOS gene expression between the diabetic + ECs and the aged control groups (p > 0.05) (Fig. 4a, b). Serum levels of endothelin and angiotensin II were significantly higher (17.38 ± 2.18 and 193.61 ± 21.19 pg/ml vs 8.49 ± 1.12 and 69.53 ± 11.27 pg/ml respectively, p < 0.05) in the aged diabetic rats when compared to the aged control group. Both serum endothelin and angiotensin II were significantly lower in the aged diabetic + ECs rats (9.27 ± 0.07 and 73.93 ± 12.42 pg/ml respectively), when compared to the aged diabetic rats group. There was no significant difference in serum endothelin and angiotensin II levels between the diabetic + ECs and the aged control groups (p > 0.05) (Fig. 4c, d).

**Fig. 5:**
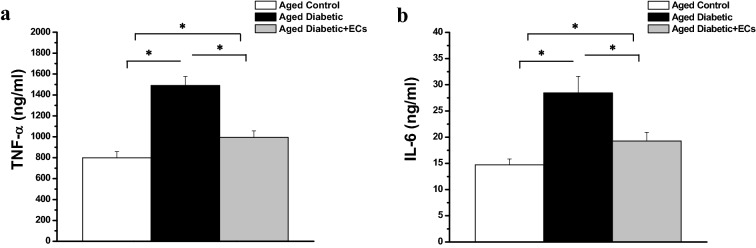
MSCs-derived ECs possess anti-inflammatory properties **a** Serum level of tumor necrosis factor alpha (TNF-α) in aged control (white column), aged diabetic (black column) and aged diabetic + ECs treated (grey column) groups. **b** Serum level of interleukin 6 (IL-6) in aged control (white column), aged diabetic (black column) and aged diabetic + ECs treated (grey column) groups. (*Significant (p < 0.05), ns = non significant. Number of rats = 10/group)

Serum TNF-α was significantly higher in the aged diabetic rats when compared to the aged control group (1489.74 ± 86.81 vs 798.23 ± 58.26 ng/ml, p < 0.05). Serum TNF-α alpha was significantly lower in the aged diabetic + ECs rats when compared to the aged diabetic group (993.92 ± 62.83 ng/ml, p < 0.05). Serum IL-6 was significantly higher in the aged diabetic when compared to the aged control rats (26.79 ± 2.86 vs 13.72 ± 1.11 nM/ml, p < 0.05), while it was significantly lower in the aged diabetic + ECs rats when compared to the aged diabetic group (18.23 ± 1.37 nM/ml, p < 0.05). However, serum levels of TNF-α and IL-6 were still significantly higher in aged diabetic + ECs when compared to aged control rats (p < 0.05) (Fig. 5a, b).

**Fig. 6:**
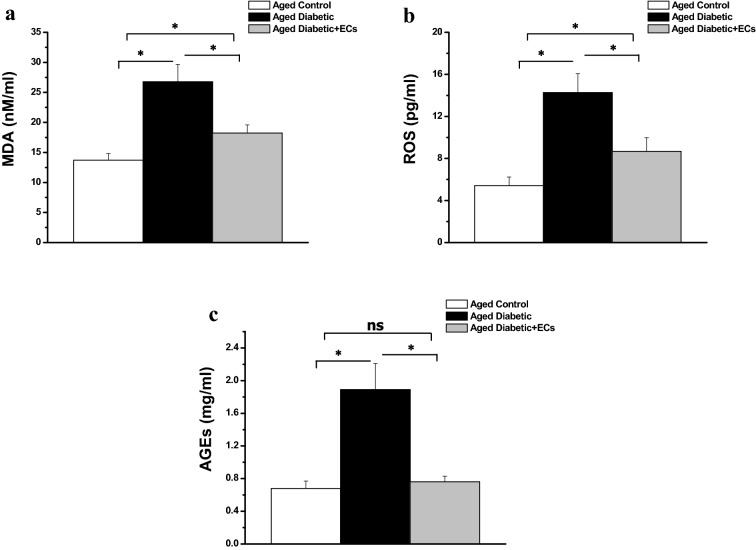
Antioxidant effects of MSCs-derived ECs. **a** Serum level of malondialdhyde (MDS) in aged control (white column), aged diabetic (black column) and aged diabetic + ECs treated (grey column) groups. **b** Serum level of reactive oxygen species (ROS) in aged control (white column), aged diabetic (black column) and aged diabetic + ECs treated (grey column) groups. **c** Serum level of advanced glycation end products (AGEs) in aged control (white column), aged diabetic (black column) and aged diabetic + ECs treated (grey column) groups. (*Significant (p < 0.05), ns = non significant. Number of rats = 10/group)

As shown in Fig. 6, serum levels of MDA, ROS and AGEs in aged diabetic rats were significantly higher when compared to the aged control group (26.79 ± 2.86, 14.27 ± 1.79 and 1.89 ± 0.32 vs 13.72 ± 1.11 nM/ml, 5.42 ± 0.81 pg/ml and 0.68 ± 0.09 mg/ml respectively, p < 0.05). Serum levels of MDA, ROS and AGEs were significantly lower in the aged diabetic + ECs rats (18.23 ± 1.37 nM/ml, 8.68 ± 1.29 pg/ml and 0.76 ± 0.07 mg/ml respectively, p < 0.05), when compared to the aged diabetic group. However, serum MDA ROS levels remained significantly higher in the aged diabetic + ECs group when compared to the corresponding values in the aged control group (p < 0.05). There was no significant difference in serum AGEs level between the diabetic + ECs and the aged control groups (p > 0.05).

**Fig. 7:**
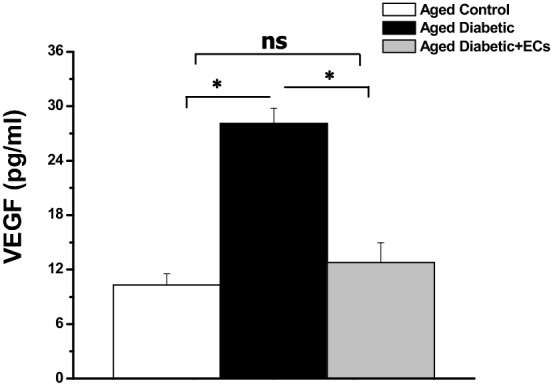
Anti-angiogenic effect of MSCs-derived ECs. Serum level of vascular endothelial growth factor (VEGF) in aged control (white column), aged diabetic (black column) and aged diabetic + ECs treated (grey column) groups. (*Significant (p < 0.05), ns = non significant. Number of rats = 10/group)

Serum VEGF level was significantly higher in the aged diabetic rats when compared to the aged control rats (28.1 ± 1.68 vs 10.3 ± 1.25 pg/ml, p < 0.05). While serum VEGF was significantly lower in the aged diabetic + ECs rats when compared to the aged diabetic group (12.8 ± 2.17 pg/ml, p < 0.05). There was no significant difference in serum VEGF levels when comparing the diabetic + ECs and the aged control rats (p > 0.05) (Fig. 7).

## Discussion

Aging remains a major risk factor for the development of CVD which is partially attributable to the progress of vascular endothelial dysfunction. Endothelial dysfunction is indicated by reduced peripheral artery endothelium-dependent dilation (EDD) in response to either chemical (acetylcholine) or mechanical (intravascular shear) stimuli [19]. Even under tight glycemic control, diabetes mellitus is associated with increased risk of CVD. Substantial clinical and experimental evidence suggest that diabetes causes endothelial dysfunctions, and thereby, restricts the vascular endothelium anti-atherogenic capacity [3]. Unsurprisingly, diabetes in elderly presents a growing public health burden. Indeed, older adults represent one of the fastest growing segments of the diabetes population and the numbers are anticipated to grow significantly over the next decades [20]. MSCs encompass all the criteria of authentic stem cells; they have the capacity to differentiate into multiple cell linages in vitro under controlled conditions. MSCs can differentiate into endothelial cells, osteocytes, chondrocytes, adipocytes and skeletal muscle cells [12, 21, 22]. In vivo, MSCs had the ability to differentiate into smooth muscle and endothelial cells increasing the vascularity and recovering the cardiac function in a canine chronic ischemia model [23]. Herein, we show a potential role of endothelial cells differentiated from Wharton’s Jelly-derived mesenchymal stem cells in attenuating altered vascular functions in old diabetic rats.

MSCs, also known as multipotent mesenchymal stromal cells, are self-regenerating cells that are found in apparently all postnatal organs and tissues [24]. In the present study, freshly isolated WJ-MSCs progressively demonstrated fibroblast-like appearance in the first 10–15 days of seeding, and required 2–4 weeks until fibroblast-like adherent cells reached 80–90% confluence. The cultured WJ-MSCs were expressing CD44 but not CD34. CD44 is a mesenchymal lineage specific marker, while CD34 is a haematopoietic linage endothelial marker [25]. Cells were then differentiated into ECs by using VEGF-enriched media. As a proof of differentiation, the VEGF-treated WJ-MSCs cells showed positive expression of CD34. It was reported before that culturing MSCs in an endothelial growth supplements-enriched medium resulted in increased expression of endothelial markers such as CD31, D34, CD105, CD144, KDR, and UEA-1 [26]. Had proven this, we then went to validate the functionality of the transformed ECs in an aged diabetic rat model.

In the present study systolic blood pressure was significantly higher, and both aortic pulse wave velocity (PWV) and renal artery resistance were significantly increased in the aged diabetic group when compared to the aged non-diabetic control group. Treatment with MSC-derived ECs resulted in substantial lowering of systolic blood pressure and decrease in PWV and renal artery resistance. The aged diabetic patients represent a distinctive subset of the population with an extraordinarily high prevalence of hypertension and CVD risk. Previous reports suggest that 67% patients with self-reported diabetes for 20 years had hypertension, and that tight blood pressure control significantly improved macro- and micro-vascular complications in diabetic patients [27]. Aging, diabetes mellitus and hypertension share common pathways including endothelial dysfunction, inflammation, oxidative stress, insulin resistance, autonomic dysfunction and alteration of the rennin angiotensin system. These pathways could overlap and influence each other and even form a vicious cycle [27–29]. It has been reported previously that arterial stiffness is increased in diabetic patients. Pulse wave velocity (PWV) is considered to be the gold-standard parameter to assess arterial stiffness [30]. Interestingly, diabetic patients with abnormal PWV were significantly older than diabetic patients with normal PWV [31]. This could imply that aging may exacerbate cardiovascular events in diabetic patients. Diabetes mellitus and hypertension coexistence significantly promotes chronic kidney disease (CKD). Diabetic nephropathy is mainly secondary to atherosclerosis of the renal arteries, as well as microangiopathy of the glomerular capillaries, afferent and efferent arterioles. Previous reports have shown that renal artery resistance is increased in type II diabetes mellitus [32]. Accumulating data support the possible potential role of stem cells in cardiovascular disorders including diabetic cardiovascular events. MSCs were reported to differentiate into ECs and vascular smooth muscle cells in vivo, and to improve cardiac function in a chronic ischemia model [23].

Endothelial dysfunction, oxidative stress and inflammation are key risk factors for CVD in aging and diabetes. Even in the absence of clinical CVD or major risk factors for CVD, vascular endothelial dysfunction develops and can be observed in older compared with young adult humans or animal models [33]. However, the interaction between the three mechanisms is imprecise. Due to the complexity of the aetiology of endothelial dysfunction, it was necessary to improve the diagnostic accuracy by measuring multiple bioactive molecules that links endothelial dysfunction to the altered inflammatory and oxidative status in aged diabetics.

In the present study there was a significant reduction in both tissue NO and eNOS levels, while there was significant elevation of serum ET-1 and ANG-II in aged diabetic rats that could be greatly countered by treatment with ECs. A balanced release of endothelial-derived relaxing and contracting factors exists under physiological conditions. Endothelial dysfunction represents an imbalance in endothelial production of mediators that regulate vascular tone, platelet aggregation, coagulation and fibrinolysis, of which vascular tone has gained much attention. Reduced availability of NO has been strongly linked to the impairment of endothelial dependent dilatation (EDD). eNOS catalyses the synthesis of NO from l-arginine [19]. NO maintains blood vessels in a constant state of vasodilation, while ET-1 and ANG-II have the opposite effect causing vasoconstriction. Endothelial dysfunction results from the imbalance between vasodilation and vasoconstriction, and the progress of atherosclerosis and cardiovascular events is much attributable to this imbalance [34]. Both eNOS activity and expression are directly reduced by IL-6. IL-6 also increases vascular superoxide production, which inactivates NO promptly resulting in the reduction in NO bioavailability [35]. In other words, IL-6 can directly alter NO synthesis and bioavailability. TNF-α dependent Endothelial dysfunction in type II diabetes was linked to over production of ROS and a decrease in NO bioavailability [36]. Moreover, TNF-α interacts with IL-6 resulting in exacerbation of oxidative stress and reduction in eNOS phosphorylation. This interaction has been shown to contribute strongly to endothelial dysfunction in type II diabetes [37]. Furthermore, IL-6 induced oxidative stress and endothelial dysfunction was attributable to the increase in the expression of vascular angiotensin II type 1 (AT1) receptors. The interaction between IL-6 and the renin-angiotensin system (RAS) represents a key pathophysiological mechanism in the progress of CVS events [38]. In vivo studies had shown that the upregulation of TNF-α may contribute to endothelial dysfunction-induced enhanced vasoconstriction by altering the vascular angiotensin II system. TNF-α inhibition resulted in reduced vasoconstriction of mesenteric arteries which was accompanied by decrease in vascular expression of angiotensin II, angiotensin-converting enzyme, and AT1 receptors [39]. Consequently, we could reason that the treatment with ECs has attenuated the cytokines-induced alteration in vascular tone. We could also suggest that the improvement in SBP, aortic artery PWV and renal artery resistance in the ECs treated aged diabetic rats could be attributable to the anti-inflammatory properties of ECs. Although previous reports had shown that MSCs possess anti-inflammatory properties [40], to our knowledge this is the first report that demonstrates in vivo the anti-inflammatory properties of MSC-derived ECs.

In the present study, there was significant increase in the oxidative stress hallmarks; reactive oxygen species (ROS), malondialdhyde (MDA) and advanced glycation end products (AGEs) in the aged diabetic group when compared to the aged non-diabetic group. Oxidative stress markers were significantly reduced following ECs treatment. Accumulating data suggest that oxidative stress may underlie diabetic complications as well as aging. Indeed, the major cause of morbidity and mortality in diabetics is the long-term vascular complications [41]. Uncontrolled production of ROS has serious impact on the progression of diabetes mellitus. Hyperglycaemia results in overproduction of ROS, which in turn can damage the endothelial cells of small and large vessels, as well as the myocardium [42] MDA level in diabetic patients has been reported to be elevated in plasma, serum, and many others tissues as a results of peroxidative injury. Essentially, disturbance of lipid structure and metabolism has been reported particularly in patients with diabetic vascular complications [43]. AGEs are produced by irreversible glycoxidation of fructoselysine and correlate directly with the vascular and renal complications in diabetes mellitus. These carbohydrate-derived protein oxidation products are more abundant in diabetic patients than in age-matched control subjects [44]. AGEs formation has deleterious effect on both lipids and proteins resulting in lipid peroxidation, platelet activation and eventually rapid progression of cardiovascular complications [41]. Interestingly, restriction of dietary AGEs resulted in significant decrease in serum levels of MDA and TNF-α [45], reflecting a close relation between oxidative stress and inflammation in diabetes mellitus. Interestingly, MSCs transplantation prevented the decrease in the levels of the natural antioxidant protein glutathione (GSH) as well as the superoxide dismutase (SOD) enzyme activity suggesting an antioxidant property of MSC [46]. Interestigly, bone marrow-derived endothelial progenitor cells (EPCs) were found to attenuate cerebral ischemia and reperfusion injury via antioxidant, anti-inflammatory and anti-apoptotic effects [47].

Herein, we demonstrated that the serum levels of the vascular endothelial growth factor (VEGF) were significantly higher in the diabetic aged group when compared to the control group. Treatment with ECs resulted in significant reduction in serum VEGF levels. It is generally accepted that VEGF is the most potent proangiogenic growth factor. VEGF increases vascular permeability, activates endothelial cells and is implicated in diabetic endothelial dysfunction and microvascular complications [48]. Accumulating data demonstrated that the circulating levels of VEGF were higher in diabetic patients when compared to the healthy control individuals, with a noticeable positive correlation of plasma VEGF levels with diabetic microvascular complications; diabetic nephropathy, retinopathy and peripheral neuropathy [48–50]. Diabetes itself, endothelial dysfunction or vascular injury induced hypoxia may increase the production of VEGF. Interestingly, angiotensin converting enzyme (ACE) inhibitors-treated diabetic patients had lower vitreous levels of VEGF, indicating that angiotensin II may also contribute to the elevated levels of VEGF seen in diabetes mellitus [51]. This could be one of the first reports to demonstrate a direct relation between transplanted ECs and endogenous VEGF levels. The decreased inflammation and oxidative stress and the improved endothelial function following treatment with ECs may explain the decrease in VEGF levels seen in the present study.

## Conclusion

Aged diabetics represent a serious societal health problem due to their vulnerability to cardiovascular events. hWMSCs are a reliable source for MSCs that can be differentiated into ECs. When injected into aged diabetic rats, differentiated ECs reduced systolic blood pressure, ameliorated endothelial dysfunction, and demonstrated potent anti- inflammatory and antioxidant properties. Taken together, our data reports a previously unpublished potential role of MSC-derived ECs in the management of diabetes in elderly. MSC-derived ECs countered the triad of endothelial dysfunction, inflammation and oxidative stress that underlies aging, diabetes and hypertension.

## Data Availability

Data supporting findings are presented within the manuscript.

## Abbreviations

AGEs: Advanced glycation end products
AGN-II: Angiotensin II
DMEM: Dulbecco’s modified eagle’s medium
ECs: Endothelial cells
eNOS: Endothelial NO synthase
ET-1: Endothelin-1
iNOS: Inducible NO synthase
IL-6: Interleukin 6
MDA: Malondialdehyde
MSCs: Mesenchymal stem cells
NO: Nitric oxide
PWV: Pulse wave velocity
ROS: Reactive oxygen species
STP: Sodium thiopental
SBP: Systolic blood pressure
TNF-α: Tumour necrosis factor alpha
VEGF: Vascular endothelial growth factor
